# Myc-Driven Overgrowth Requires Unfolded Protein Response-Mediated Induction of Autophagy and Antioxidant Responses in *Drosophila melanogaster*

**DOI:** 10.1371/journal.pgen.1003664

**Published:** 2013-08-08

**Authors:** Péter Nagy, Ágnes Varga, Karolina Pircs, Krisztina Hegedűs, Gábor Juhász

**Affiliations:** Department of Anatomy, Cell and Developmental Biology, Eötvös Loránd University, Budapest, Hungary; Zentrum für Molekulare Biologie der Universität Heidelberg, Germany

## Abstract

Autophagy, a lysosomal self-degradation and recycling pathway, plays dual roles in tumorigenesis. Autophagy deficiency predisposes to cancer, at least in part, through accumulation of the selective autophagy cargo p62, leading to activation of antioxidant responses and tumor formation. While cell growth and autophagy are inversely regulated in most cells, elevated levels of autophagy are observed in many established tumors, presumably mediating survival of cancer cells. Still, the relationship of autophagy and oncogenic signaling is poorly characterized. Here we show that the evolutionarily conserved transcription factor Myc (dm), a proto-oncogene involved in cell growth and proliferation, is also a physiological regulator of autophagy in *Drosophila melanogaster*. Loss of Myc activity in null mutants or in somatic clones of cells inhibits autophagy. Forced expression of Myc results in cell-autonomous increases in cell growth, autophagy induction, and p62 (Ref2P)-mediated activation of Nrf2 (cnc), a transcription factor promoting antioxidant responses. Mechanistically, Myc overexpression increases unfolded protein response (UPR), which leads to PERK-dependent autophagy induction and may be responsible for p62 accumulation. Genetic or pharmacological inhibition of UPR, autophagy or p62/Nrf2 signaling prevents Myc-induced overgrowth, while these pathways are dispensable for proper growth of control cells. In addition, we show that the autophagy and antioxidant pathways are required in parallel for excess cell growth driven by Myc. Deregulated expression of Myc drives tumor progression in most human cancers, and UPR and autophagy have been implicated in the survival of Myc-dependent cancer cells. Our data obtained in a complete animal show that UPR, autophagy and p62/Nrf2 signaling are required for Myc-dependent cell growth. These novel results give additional support for finding future approaches to specifically inhibit the growth of cancer cells addicted to oncogenic Myc.

## Author Summary

The evolutionarily conserved transcription factor Myc promotes protein synthesis, cell growth and cancer progression through incompletely understood mechanisms. In this work, we show that forced expression of Myc induces the accumulation of abnormal proteins leading to unfolded protein responses (UPR), presumably by overloading the protein synthetic capacity of cells in Drosophila. UPR then results in autophagy-mediated breakdown and recycling of cytoplasmic material, and at the same time, to activation of antioxidant responses in these cells. Blocking the UPR stress signaling, autophagy and antioxidant pathways genetically, or by feeding larvae an autophagy-inhibiting drug, prevents overgrowth of Myc-expressing cells, but these treatments do not affect the growth of control cells in the same tissues. These results, together with recent reports in mammalian cancer models, suggest that drugs targeting UPR, autophagy and antioxidant responses may specifically inhibit cancer cell proliferation driven by oncogenic Myc.

## Introduction

The balance of anabolic and catabolic processes determines net changes in cell size. An increased ratio of biosynthetic versus degradative activity results in cell growth. Activation of growth-promoting signaling pathways such as the class I PI3K (phosphatidylinositol 3-kinase), AKT and TOR (target of rapamycin) kinase systems increases protein translation and inhibits autophagy, an *Atg* gene dependent pathway in eukaryotic cells that captures proteins, lipids and organelles in double-membrane autophagosomes, followed by lysosomal degradation and recycling [1]. In addition, we and others have shown previously that genetic activation of autophagy reduces cell size in flies and mammals, and autophagy-deficient cells have a net growth advantage during long-term starvation in Drosophila melanogaster larvae [2], [3].

However, the role of autophagy in cell growth and physiology is more complex. Low-level basal autophagy maintains protein and organelle quality control by selectively removing unfolded proteins and damaged or superfluous organelles. Loss of basal autophagy results in gradual accumulation of toxic protein aggregates and excess or non-functional organelles, leading to pathological consequences including neurodegeneration and decreased lifespan both in Drosophila and mice [4]–[6]. Autophagy deficiency is associated with increased production of reactive oxygen species, proteotoxicity and accelerated tumorigenesis [7]. Autophagy is upregulated in response to starvation and stress to promote survival by recycling dispensable intracellular components. Large-scale autolysosomal degradation provides building blocks that fuel biosynthetic and metabolic pathways under these conditions. Elevated levels of autophagy are also observed in many established cancers [6]–[8]. While the underlying genetic changes are still poorly characterized, autophagy induction has been suggested to sustain the altered metabolism and survival of various cancer cells. These reports altogether indicate that autophagy has a context-dependent role in the regulation of cell growth.

Myc is required for proper expression of genes involved in various processes including cell growth and proliferation [9]–[11]. It forms an evolutionarily conserved heterodimeric transcription factor with its binding partner Max. Myc-Max complexes show high affinity binding to E-box sequences in the promoters or introns of their target genes to increase their transcription rate. In addition, Myc exhibits lower affinity binding to additional promoter sequences to regulate a much larger set of genes. Myc is a classical oncogene deregulated in most tumors, although the mechanisms of how it promotes overgrowth of cancer cells are incompletely understood [9]–[12]. We decided to explore potential connections between Myc, autophagy, and Myc-induced overgrowth in the genetic model organism Drosophila.

## Results

**Figure 1:**
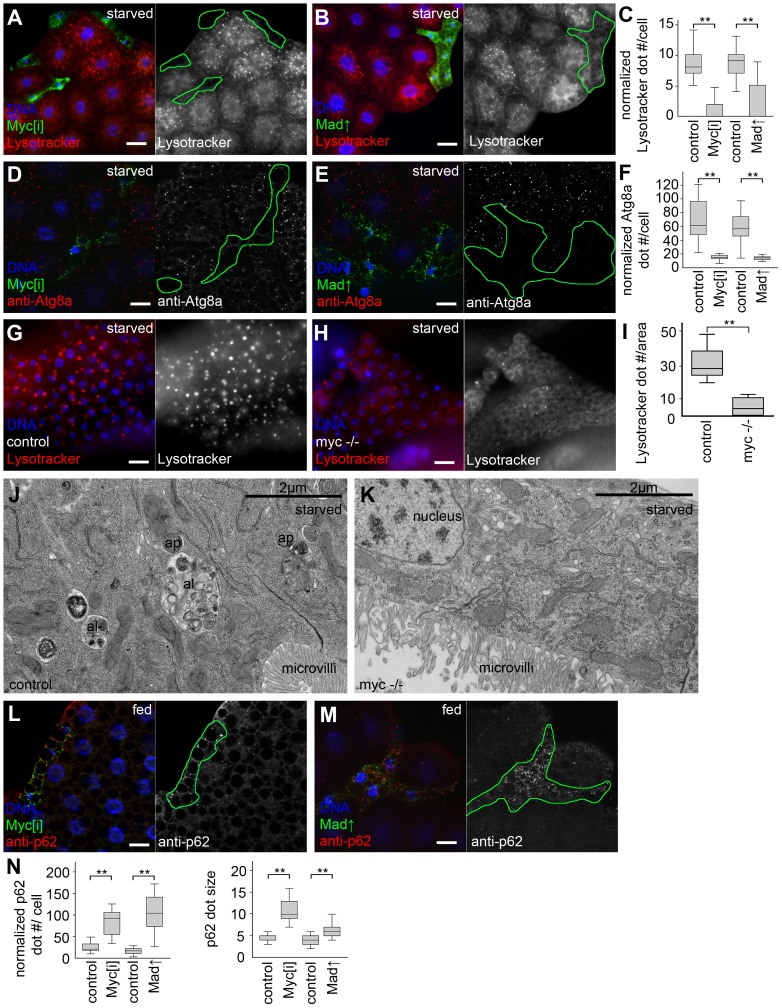
Myc is necessary for starvation-induced autophagy in Drosophila. (A) *Myc* depletion in LAMP1-GFP marked fat body cell clones inhibits starvation-induced punctate Lysotracker staining, compared to surrounding control cells that do not express GFP. Mid-L3 stage larvae were analyzed in all fat body experiments unless noted otherwise. (B) Overexpression of Mad also blocks Lysotracker dot formation in cells marked by LAMP1-GFP. (C) Quantification of data presented in panels A and B, n = 7 for all genotypes. (D) Starvation-induced punctate endogenous Atg8a staining is inhibited in *Myc* RNAi cells marked by LAMP1-GFP expression, compared to neighboring control tissue. (E) Forced expression of Mad also decreases Atg8a dot formation. (F) Quantification of data presented in panels D and E, n = 6 for all genotypes. (G) Starvation leads to the formation of Lysotracker-positive autolysosomes in fat body cells of control L1 stage larvae. (H) A near-complete block of Lysotracker puncta formation is seen in *Myc* null mutant L1 stage larvae. (I) Quantification of data presented in panels G and H. (J) Double-membrane autophagosomes (ap) and dense autolysosomes (al) degrading sequestered cytoplasmic cargo are observed in midgut cells of control L1 stage larvae. (K) No autophagic structures are seen in midgut cells of L1 stage *Myc* null mutant larvae. (L) The selective autophagy cargo p62 accumulates in *Myc* RNAi cells (marked by LAMP1-GFP expression) compared to surrounding control tissue in well-fed animals. (M) Mad overexpression also leads to large-scale accumulation of p62 aggregates. (N) Statistical evaluation of data presented in panels M and N, n = 7 for all genotypes. Scalebars in A, B, D, E, G, H, L, M equal 20 µm. Statistically significant differences are indicated, ** p<0.01.

Depletion of *Myc* (also known as *dm*) by expression of a transgenic RNAi line, or overexpression of its antagonist Mad (also known as Mnt) in GFP-marked clones reduced the size of polyploid fat body cells in a cell-autonomous manner, as described earlier [13], [14]. Lysotracker red is an acidophilic dye commonly used to label digesting autolysosomes in Drosophila [2], [4], [15]–[19]. Myc activity was required for starvation-induced formation of Lysotracker-positive punctae, as GFP-positive *Myc* RNAi or Mad overexpressing cells had fewer dots compared to surrounding control fat tissue (Figure 1A–C, see also Table S1 for statistical analyses of these and all subsequent data). Activated, lipidated Atg8a is covalently bound to autophagosomes, and is rapidly degraded upon their fusion with lysosomes, so analyzing endogenous Atg8a-positive puncta is widely used to follow the generation of early autophagic structures [16], [19], [20]. Silencing of *Myc* or overexpression of Mad in GFP-positive cell clones also interfered with starvation-induced punctate endogenous Atg8a labeling compared to control cells in starved larvae (Figure 1D–F). Similarly, *Myc* null mutant larvae showed impaired starvation-induced autophagy of the fat body and midgut, based on Lysotracker staining and transmission electron microscopy (Figure 1G–K). p62 (also known as Ref2P, for refractory to sigma P) is a constitutively expressed selective autophagic cargo. Impaired autophagy leads to stabilization of p62, so analyzing the levels of p62 aggregates is a standard assay for estimating autophagic degradation [16], [17], [19], [21]. Loss of Myc activity in GFP-marked cells in well-fed larvae cell-autonomously increased both the number and size of p62 aggregates (Figure 1L–N), suggesting basal autophagy defects. These results established that *Myc* is a physiological regulator of autophagy.

**Figure 2:**
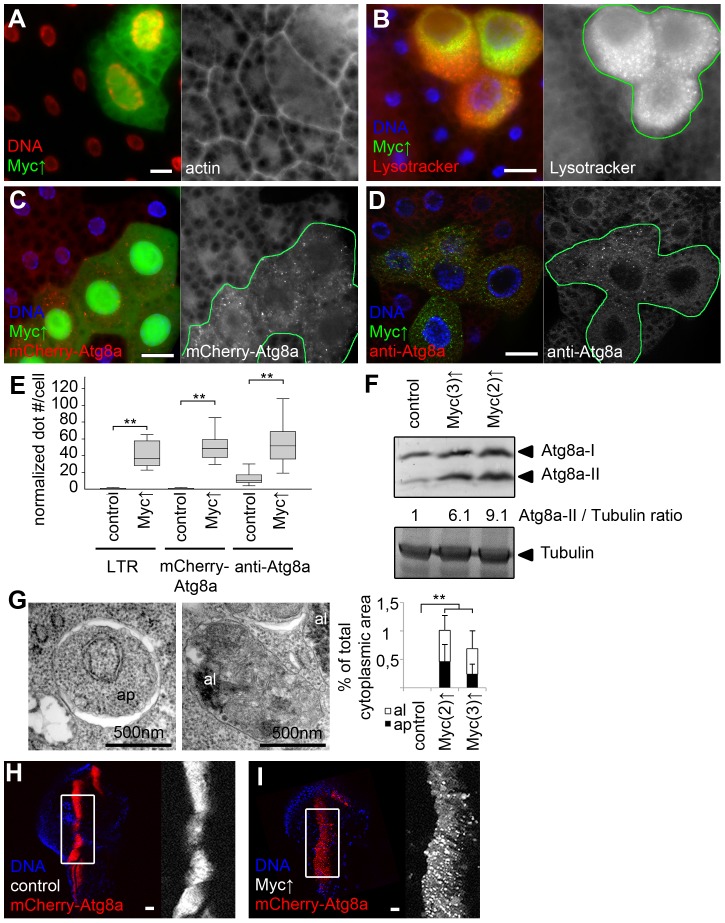
Myc overexpression induces autophagy in Drosophila. (A) Myc overexpression in GFP-marked fat body cell clones leads to cell-autonomous overgrowth. (B–D) Enhanced punctate Lysotracker staining (B), mCherry-Atg8a dot formation (C) and endogenous Atg8a labeling (D) is observed in Myc-overexpressing fat body cell clones (marked by expression of LAMP1-GFP in panels B and D and by GFP in panel C), when compared to surrounding control tissue in well-fed larvae. (E) Quantification of data presented in B–D, n = 7 for all genotypes. (F) Fat body-specific expression of Myc from transgenes located on chromosome 2 or 3 increase the amount of activated Atg8a-II compared to control fat bodies of well-fed larvae. Numbers indicate the level of lipidated Atg8a-II relative to the loading control Tubulin in different genotypes, as determined by densitometric evaluation. (G) Fat body-specific overexpression of Myc induces formation of typical double-membrane autophagosomes (ap) and autolysosomes that contain cytoplasmic material in various stages of degradation (al), n = 5 for all genotypes. (H, I) Myc overexpression leads to punctate mCherry-Atg8a labeling (I) and increased expression area in the *patched* domain of wing discs compared to control discs (H). Scalebars in A–D and H–I equal 20 µm. Statistically significant differences are indicated, ** p<0.01.

Overexpression of Drosophila Myc in GFP-marked fat body cell clones increased cell growth compared to neighboring control cells, as described previously (Figure 2A) [14], [22]. Myc overexpression activated autophagy in fat body cells of well-fed Drosophila larvae in a cell-autonomous manner, based on the induction of Lysotracker-positive autolysosomes, punctate mCherry-Atg8a that labels both autophagosomes and autolysosomes as mCherry remains fluorescent in lysosomes [16], [17], [19], [20], [23], and increased formation of endogenous Atg8a-positive autophagosomes in GFP-positive cells compared to neighboring control cells (Figure 2B–E). Determining the levels of autophagosome-associated, lipidated Atg8a-II relative to loading control in western blot experiments is a standard biochemical assay to analyze autophagy [16], [19], [24]. Myc overexpression in whole larval fat bodies indeed elevated the amounts of Atg8a-II relative to Tubulin (Figure 2F). Electron microscopy confirmed the presence of autolysosomes containing cytoplasmic material in various stages of degradation, and double-membrane autophagosomes containing undigested cargo in Myc overexpressing cells (Figure 2G, Figure S1). Similarly, forced expression of Myc in a stripe of cells in wing imaginal discs increased the size of this expression domain and enhanced mCherry-Atg8a dot formation (Figure 2H, I), indicating autophagy induction in these diploid cells.

**Figure 3:**
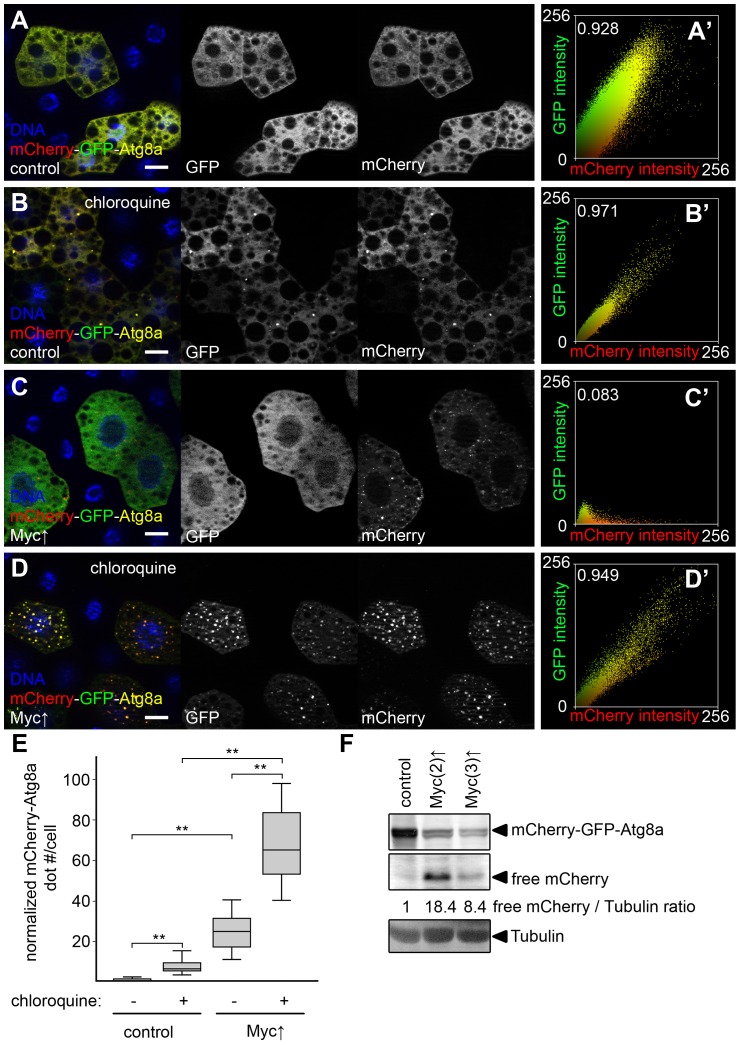
Myc increases autophagic flux. (A) Practically no mCherry-GFP-Atg8a dots are seen in control fat body cells of well-fed larvae. (B) Dots positive for both mCherry and GFP appear in control cells upon feeding larvae chloroquine, a drug that blocks lysosomal degradation. (C) Overexpression of Myc results in the formation of puncta that are mostly mCherry-positive. (D) Chloroquine treatment strongly increases the number of mCherry punctae in Myc overexpressing cells. Note that dots are now positive for GFP as well. (A′–D′) Fluorescent intensity and colocalization profiles of mCherry and GFP channels from panels A–D. Pearson correlation coefficients shown in A′–D′ indicate that the correlation of the mCherry and GFP pixel intensity is low in panel C but high in panels A, B, and D. (E) Quantification of data in panels A–D, n = 15–30 per genotype/treatment. Myc expression in fat body cells strongly induces mCherry puncta formation compared to control cells. Chloroquine treatment increases dot numbers both in control and Myc overexpressing cells. (F) Autophagic degradation-dependent conversion of mCherry-GFP-Atg8a to free mCherry is increased by overexpression of Myc in fat body cells. Numbers indicate the level of free mCherry relative to the loading control Tubulin in different genotypes, as determined by densitometric evaluation. Scalebars in A–D equal 20 µm. Statistically significant differences are indicated, ** p<0.01.

To further characterize how Myc overexpression regulates the multi-step process of autophagy, we used a double tagged reporter transgene mCherry-GFP-Atg8a, a common tool for measuring autophagic flux (the rate of autophagic degradation) [16], [19], [25], [26]. This reporter molecule is selectively bound to autophagosomes, followed by its transport to autolysosomes where GFP is quenched rapidly but mCherry fluorescence is retained. Autophagosomes are positive for both mCherry and GFP, while autolysosomes only show mCherry fluorescence in control cells undergoing autophagy [16], [19], [25], [26]. Practically no dots were observed in control fat body cells of well-fed larvae using this reporter (Figure 3A), indicating normally low basal autophagy levels. The few dots we saw were mostly mCherry-positive (13% of mCherry dots were also positive for GFP, 10/77 dots counted). Overexpression of Myc led to the formation of numerous punctae that were also mostly positive for mCherry only (9.2% colocalization with GFP, 227/2,459 dots counted, Figure 3C).

Chloroquine is a small molecule drug that neutralizes the normally acidic lysosomal pH, and thus inhibits all lysosomal degradation routes including autophagy and endocytosis [8], [16]. Chloroquine treatment is often used to distinguish autophagy induction from impaired autolysosomal degradation: both conditions can result in increased numbers of autophagic structures, but these numbers further increase in the presence of chloroquine if autophagic flux is normal, while chloroquine has no effect if degradation is already impaired [16]. Feeding larvae chloroquine led to the induction of dots in control cells that were positive for both mCherry and GFP (98.8% colocalization, 327/331 dots counted, Figure 3B, E), indicating that low-level basal autophagic degradation was inhibited by this treatment, and autolysosomes accumulated unable to digest their cargo. Chloroquine treatment of larvae with Myc-overexpressing fat body cell clones also increased mCherry dot numbers compared to non-treated cells overexpressing Myc, and punctate mCherry-positive structures this time also contained GFP (99.2% colocalization, 4,361/4,394 dots counted, Figure 3D, E). Importantly, the number of mCherry-GFP-Atg8a positive structures was also much higher in chloroquine-treated Myc overexpressing cells than in chloroquine-treated control cells (Figure 3E), altogether suggesting that Myc promotes both the induction and degradation steps of autophagy.

Autophagic flux can also be analyzed using these fluorescently tagged Atg8a reporters in western blots, as Atg8a itself is rapidly broken down in autolysosomes, resulting in autophagic degradation-dependent generation of free mCherry or GFP [3], [16]. Again, Myc overexpression in whole fat bodies strongly increased autolysosomal conversion of mCherry-GFP-Atg8a to free mCherry relative to the loading control Tubulin (Figure 3F).

**Figure 4:**
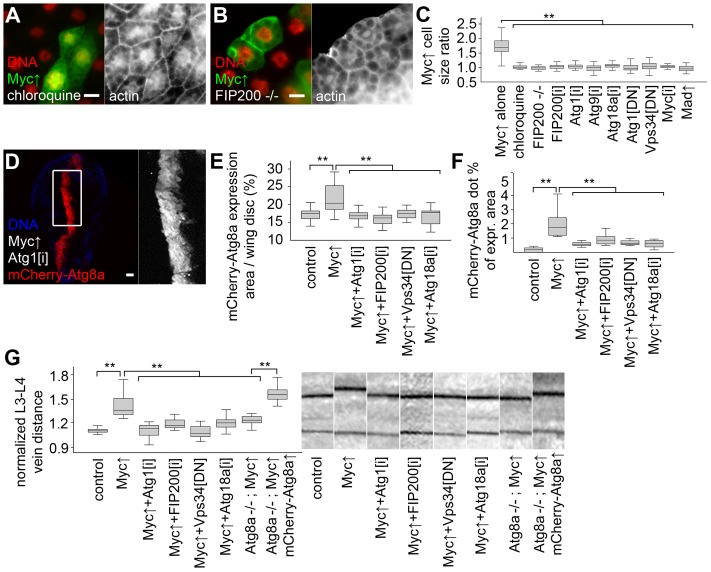
Autophagy is required for Myc-induced overgrowth. (A, B) Feeding larvae a chloroquine-containing diet (A) or null mutation of *FIP200* (B) blocks the increased growth of Myc overexpressing cells, as the size of these GFP-positive cells is now similar to neighboring control cells. (C) Statistical evaluation shows that chloroquine treatment or blocking autophagy by null mutation of *FIP200*, depletion of *FIP200, Atg1, Atg9, Atg18a*, and expression of dominant-negative (DN) Atg1 and Vps34 prevents overgrowth of Myc-expressing fat body cells compared to surrounding control cells to a similar extent as *Myc* RNAi or overexpression of Mad. n = 8–11 per genotype. (D) Knockdown of *Atg1* blocks Myc-induced overgrowth and punctate mCherry-Atg8a labeling in wing imaginal discs. (E, F) Quantification shows that inhibition of *Atg1*, *FIP200*, *Vps34* or *Atg18a* blocks Myc-induced increases of mCherry-Atg8a expression area (E; n = 10–25 per genotype) and Atg8a dot formation (F; n = 10–13 per genotype) in wing discs. (G) Myc expression in the *patched* domain increases L3–L4 wing vein distance relative to L4–L5 vein distance, which is inhibited by knockdown of *Atg1, FIP200, Atg18a*, expression of dominant-negative Vps34, or null mutation of *Atg8a*. Expression of mCherry-Atg8a restores Myc-induced increased vein distance in *Atg8a* mutants. n = 16–26 per genotype. Scalebars in A, B, D equal 20 µm. Statistically significant differences are indicated, ** p<0.01.

We next evaluated the role of autophagy in supporting the increased growth of Myc-expressing cells. Myc-induced overgrowth of GFP-positive clones of fat body cells relative to neighboring control cells was completely suppressed by chloroquine treatment (Figure 4A, C). Chloroquine was also able to suppress cellular overgrowth induced by two copies of overexpressed Myc (Figure S2A–C). Null mutation of *FIP200*, or blocking the function of other core *Atg* genes that specifically inhibit autophagic breakdown also completely suppressed the Myc-induced overgrowth of GFP-positive fat cells relative to neighboring control cells, similar to *Myc* RNAi or co-overexpression of Mad (Figure 4B, C, Figure S2D–K). Similarly, inhibition of autophagy blocked increases in Myc-overexpressing domain size and punctate mCherry-Atg8a labeling in wing imaginal discs (Figure 4D–F, Figure S2M–O). Myc expression in this domain of developing wing discs increased L3–L4 vein distance in adult wings, which was also suppressed by the silencing of *Atg1, FIP200, Atg18a*, or by blocking Vps34 (Figure 4G, Figure S2L). Importantly, Myc overexpression failed to increase L3–L4 vein distance in the absence of Atg8a, but it readily did so if mCherry-Atg8a was co-expressed in *Atg8a* null mutants (Figure 4G). Inhibition of autophagy did not reduce growth of fat body or wing intervein cells in the absence of forced Myc expression in well-fed animals (Figure S2P, Q), similar to our earlier reports [2], [4], [15]. Autophagy-dependent overgrowth of Myc expressing cells was also obvious using a different wing expression system (Figure S3) [27].

**Figure 5:**
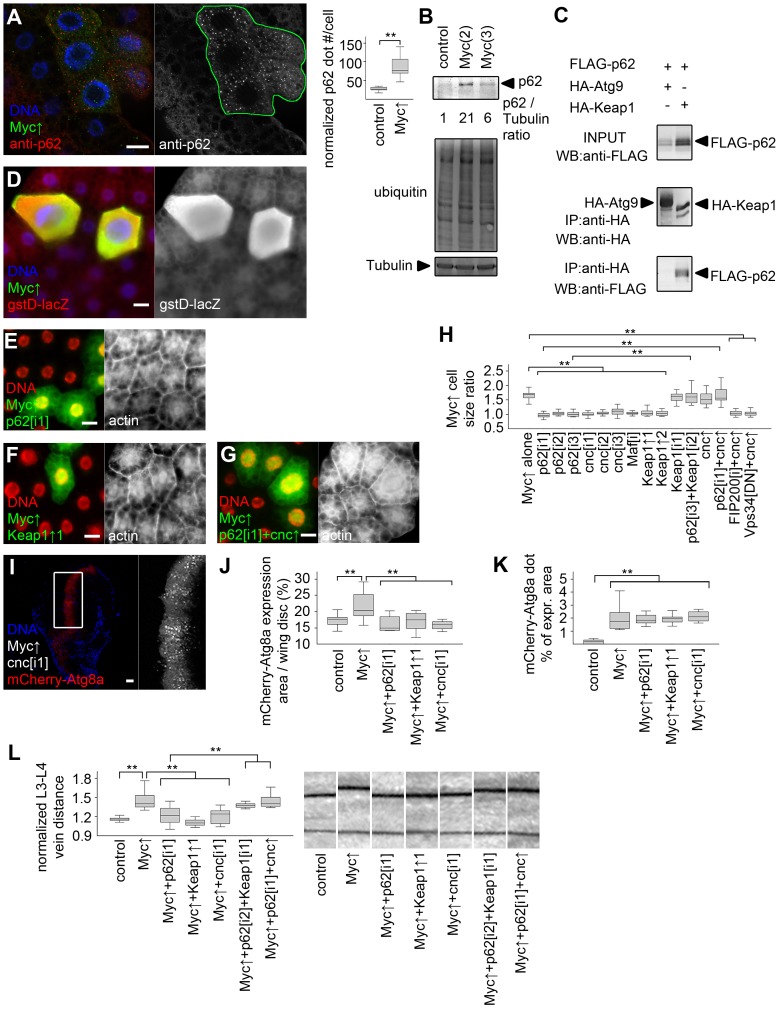
Activation of cnc/Nrf2 is required for Myc-induced overgrowth. (A) p62 aggregates accumulate in Myc-expressing cells (marked by LAMP1-GFP) relative to neighboring control cells, n = 7 for both genotypes. (B) Overexpression of Myc upregulates p62 and increases ubiquitinated protein profiles in fat bodies of well-fed larvae. Numbers indicate the level of p62 relative to Tubulin in different genotypes, as determined by densitometry. (C) HA-Keap1 strongly binds to FLAG-tagged Drosophila p62 in immunoprecipitation (IP) experiments in cultured cells, unlike HA-Atg9. WB, western blot. (D) Myc overexpression in GFP-positive cells activates the Nrf2/cnc-dependent transcriptional reporter gstD-LacZ. (E, F) Depletion of *p62* (E) or overexpression of Keap1 (F) inhibits Myc-induced overgrowth of GFP-positive cells relative to neighboring control cells. (G) Co-overexpression of cnc restores Myc-induced overgrowth of GFP-positive *p62* RNAi cells compared to surrounding cells. (H) Knockdown of *p62, cnc, Maf*, or overexpression of Keap1 prevents Myc-induced overgrowth relative to neighboring control cells. Co-overexpression of cnc or *Keap1* depletion has little effect on Myc-expressing cell size. Depletion of Keap1 restores Myc-induced overgrowth in *p62* RNAi cells, similar to overexpression of cnc. Myc-induced overgrowth is not restored by cnc overexpression in *FIP200* RNAi or dominant-negative Vps34 expressing cells. n = 8–11 per genotype. (I–K) Knockdown of *cnc* (I), *p62* or overexpression of Keap1 prevents Myc-induced increases in mCherry-Atg8a expression area of wing discs (quantifications shown in panel J; n = 10–25 per genotype) without affecting punctate mCherry-Atg8a labeling (K; n = 10–13 per genotype). (L) Myc overexpression in the *patched* domain increases L3–L4 vein distance, which is inhibited by knockdown of *p62* or *cnc*, or by co-overexpression of Keap1. *Keap1* depletion or cnc co-overexpression restores Myc-induced increased vein distance in *p62* RNAi wings. n = 8–25 per genotype. Scalebars in A, E–G, I equal 20 µm, and 40 µm in D. Statistically significant differences are indicated, ** p<0.01.

Human p62 (also known as SQSTM1 for sequestosome 1) is a multidomain adaptor protein involved in various diseases including cancer [28]. Drosophila p62 has a similar domain structure including an N-terminal PB1 (Phox and Bem1p) domain mediating its aggregation, an ubiquitin-binding UBA (ubiquitin-associated) domain, and an Atg8-interacting motif responsible for its selective degradation by autophagy [17], [21]. Myc overexpression led to the accumulation of both p62 and ubiquitinated proteins in Drosophila fat body cells (Figure 5A, B). p62 was recently shown to be required for tumorigenesis in various cancer models, and mediate persistent activation of the transcription factor Nrf2 (nuclear factor erythroid-related factor 2) [29]–[32]. Mechanistically, human p62 binds to Keap1 (kelch-like ECH-associated protein 1) by disrupting the Keap1-Nrf2 interaction, resulting in stabilization of Nrf2 and induction of antioxidant responses [31], [33]. Drosophila p62 also bound to Keap1 (Figure 5C). Consistent with these, overexpression of Myc activated the Nrf2-dependent transcriptional reporters *gstD-LacZ* and *gstD-GFP* in Drosophila fat body and wing disc cells, respectively (Figure 5D, Figure S4A, B). Knockdown of *p62* or the Drosophila *Nrf2* homologue *cnc* (cap and collar) prevented the activation of *gstD-GFP* by Myc (Figure S4C, D). This indicated that p62/Nrf2 signaling is required for Myc-induced activation of the antioxidant response reporter. Depletion of *p62* also blocked the overgrowth of Myc-expressing cell clones (marked by GFP) compared to surrounding control cells, similar to overexpression of Keap1, or silencing *cnc* or its binding partner *Maf* (Figure 5E, F, H, Figure S4E–K). Further activation of antioxidant responses by overexpression of cnc or knockdown of *Keap1* did not have much influence on overgrowth of Myc-expressing cells relative to neighboring control cells, but these genetic manipulations restored Myc-driven increased cell growth in *p62* RNAi cells (Figure 5G, H, Figure S4L–N). In contrast, Myc-induced overgrowth of GFP-positive cells relative to neighboring control cells could not be restored in *FIP200* RNAi or dominant-negative Vps34 expressing cells by co-overexpression of cnc (Figure 5H, Figure S4O, P), suggesting that Nrf2 signaling acts parallel to autophagy. Inhibition of p62/Nrf2 signaling in wing imaginal discs reduced Myc-driven cell growth without affecting punctate mCherry-Atg8a labeling (Figure 5I–K, Figure S4S, T), indicating that p62/Nrf2 signaling is dispensable for Myc-induced autophagy. Blocking antioxidant responses by knockdown of *p62* or *cnc*, or overexpression of Keap1 inhibited Myc-induced vein distance increase as well (Figure 5L). Similar to fat bodies, activation of antioxidants by silencing *Keap1* or overexpression of cnc restored increased growth in *p62* RNAi wings expressing Myc (Figure 5L), suggesting that p62 acts upstream of Nrf2 signaling in Myc-induced cell growth. Fat body cell size and wing vein distance remained mostly similar to controls upon modulation of p62/Nrf2 signaling in the absence of Myc overexpression (Figure S4Q, R).

To gain insight into the temporal regulation of autophagy and antioxidant responses by Myc, we analyzed the activation of these processes in response to its transient overexpression. Upregulation of both autophagy and antioxidant reporters became obvious by 12 hours after heat shock-mediated induction of Myc in fat body cells (Figure S5), suggesting that Myc activates these processes with similar kinetics.

**Figure 6:**
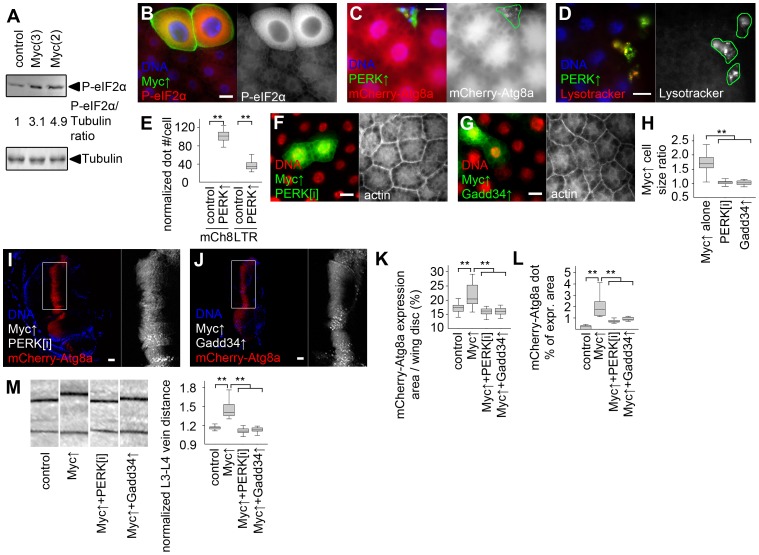
Myc-induced UPR is required for increased cell growth and autophagy. (A) Overexpression of Myc in larval fat bodies increases the level of phosphorylated eIF2α compared to controls. Numbers indicate the level of phospho- eIF2α relative to the loading control Tubulin in different genotypes, as determined by densitometric evaluation. (B) Forced expression of Myc in GFP-marked fat body cell clones increases the levels of phospho- eIF2α in a cell-autonomous manner. (C, D) Overexpression of PERK induces punctate mCherry-Atg8a (C) and Lysotracker (D) labeling in GFP-marked fat body cell clones of well-fed larvae. (E) Quantification of data presented in panels C and D, n = 7–8 per genotype. (F, G) Depletion of *PERK* (F) or co-overexpression of Gadd34 (G) prevents Myc-induced overgrowth of GFP-marked fat body cell clones relative to surrounding control cells. (H) Quantification of data presented in panels F and G, n = 9–11 per genotype. (I–L) Knockdown of *PERK* (I) or overexpression of Gadd34 (J) prevents Myc-induced increases in the mCherry-Atg8a expression area of wing discs (K; n = 10–25 per genotype) and inhibits punctate mCherry-Atg8a labeling (L; n = 10–13 per genotype). (M) Myc overexpression in the *patched* domain increases L3–L4 vein distance relative to L4–L5 vein distance in adult wings, which is inhibited by knockdown of *PERK* or by overexpression of Gadd34, n = 19–25 per genotype. Scalebars in B–D, F, G, I, J equal 20 µm. Statistically significant differences are indicated, ** p<0.01.

Increased levels of the specific autophagy cargo p62 in spite of elevated autophagy in Myc overexpressing cells suggested that p62 is also regulated independent of autophagy in this context. The unfolded protein response (UPR) in the ER and cytosol has been recently shown to upregulate p62 in cultured mammalian cells [34]. Increased ubiquitinated protein levels in Myc overexpressing fat bodies suggested that Myc induces UPR (Figure 5B). Accumulation of unfolded proteins in the ER leads to phosphorylation of eIF2α through activation of PERK (also known as PEK, for pancreatic eIF2α kinase) [35]. Indeed, Myc overexpressing fat bodies had increased levels of phosphorylated eIF2α (Figure 6A, B). Overexpression of Myc also activated the UPR-specific splicing of Xbp1, based on an in vivo reporter assay (Figure S6A, B) [36]. Genetic activation of ER stress signaling by overexpressing PERK in GFP-marked cell clones induced punctate mCherry-Atg8a and Lysotracker staining in a cell-autonomous manner in well-fed animals (Figure 6C–E), indicating that PERK activation promotes autophagy in Drosophila. Gadd34 associates with protein phosphatase 1 to dephosphorylate eIF2α, thereby antagonizing PERK signaling in Drosophila and mammals [35]. Depletion of *PERK* or overexpression of Gadd34 blocked Myc-induced overgrowth in GFP-marked fat body cell clones relative to neighboring control cells (Figure 6F–H). These genetic manipulations also prevented increases in the Myc-expressing area of wing discs, and attenuated Myc-induced autophagy based on decreased punctate mCherry-Atg8a labeling (Figure 6I–L). Silencing of *PERK* or overexpression of Gadd34 also blocked Myc-induced vein distance increase in adult wings (Figure 6M). *PERK* RNAi and Gadd34 overexpression had no effect on the growth of wild-type fat body cells, and only slightly influenced vein distance in control wings (Figure S6C, D).

## Discussion

**Figure 7:**
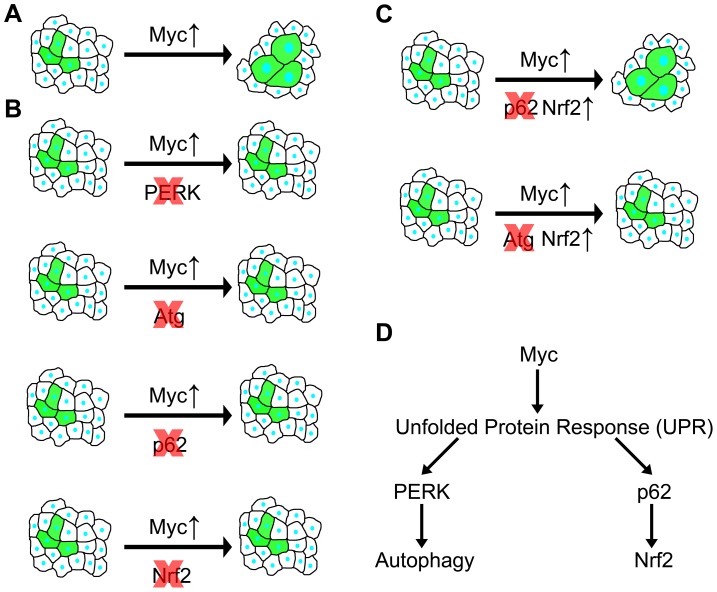
Summary of genetic interactions in the context of Myc-induced overgrowth. (A) Overexpression of Myc in GFP-positive cells leads to a cell-autonomous increase of size. (B) Genetic inhibition of PERK signaling, autophagy, p62 or Nrf2/cnc signaling prevents Myc-induced overgrowth, as GFP-positive cells are now unable to overgrow neighboring cells. (C) Overexpression of Nrf2 restores Myc-induced overgrowth in the absence of p62, while it has no effect in autophagy-deficient cells, indicating that Nrf2 acts downstream of p62 but in parallel to autophagy. (D) Overexpression of Myc induces UPR, leading to activation of PERK in the ER that triggers autophagy. At the same time, cytoplasmic UPR presumably results in accumulation of p62, which then activates Nrf2.

Previous genetic studies established that *Myc* is required for proper expression of hundreds of housekeeping genes and is therefore essential for cell growth and proliferation [9], [11]. *Myc* is a typical example of a nuclear oncogene: a transcription factor that drives tumor progression if its expression is deregulated in mammalian cells [12]. Its mechanisms of promoting cell growth are likely different in many ways from that of cytoplasmic oncogenes such as kinases encoded by *PI3K* and *AKT* genes, also frequently activated in various cancers [12]. Overexpression of these drives cell growth in Drosophila as well, but Myc also increases the nuclear∶cytoplasmic ratio in hypertrophic cells, unlike activation of PI3K/AKT signaling [14], [22]. PI3K and AKT suppress basal and starvation-induced autophagy, while their inactivation strongly upregulates this process [18]. In contrast, here we showed that both basal and starvation-induced autophagy requires *Myc*, and that overexpression of Myc increases UPR, leading to PERK-dependent induction of autophagy, and presumably to accumulation of cytoplasmic p62 that activates antioxidant responses (Figure 7A, B). Autophagy deficiency predisposes to cancer at least in part through accumulation of the selective autophagy cargo p62, resulting in activation of antioxidant responses and tumor formation [32]. Our analyses show that both of these cytoprotective pathways can be activated simultaneously, and are required in parallel to sustain Myc-induced overgrowth in Drosophila cells (Figure 7C, D).

Autophagy and antioxidant responses have been considered to act as tumor suppressor pathways in normal cells and during early stages of tumorigenesis, while activation of these processes may also confer advantages for cancer cells [7], [8]. Lack of proper vasculature in solid tumors causes hypoxia and nutrient limitation. These stresses in the tumor microenvironment were suggested to elevate UPR and autophagy to promote survival of cancer cells [37]. Our studies demonstrate that genetic alterations similar to those observed in cancer cells (that is, deregulated expression of Myc) can also activate the UPR, autophagy and antioxidant pathways in a cell-autonomous manner in Drosophila. These processes are likely also activated as a consequence of deregulated Myc expression in human cancer cells based on a number of recent reports, similar to our findings in Drosophila presented here. First, chloroquine treatment that impairs all lysosomal degradation pathways was sufficient to reduce tumor volume in Myc-dependent lymphoma models [38], [39]. Second, a related study has been published while we were preparing this manuscript, showing that ER stress and autophagy induced by transient Myc expression increased survival of cultured cells, and PERK-dependent autophagy was necessary for tumor formation in a mouse model [40]. Our data suggest that UPR-mediated autophagy and antioxidant responses may also be necessary to sustain the increased cellular growth rate driven by deregulated expression of Myc.

Myc has proven difficult to target by drugs. Myc-driven cancer cell growth could also be selectively prevented by blocking cellular processes that are required in cancer cells but dispensable in normal cells, known as the largely unexplored non-oncogene addiction pathways [41]. Previous genetic studies established that autophagy is dispensable for the growth and development of mice, although knockout animals die soon after birth due to neonatal starvation after cessation of placental nutrition [6], [42]. Tissue-specific *Atg* gene knockout mice survive and the animals are viable, with potential adverse effects only observed in aging animals [6], [32], [43]. Genetic deficiencies linked to *p62* are also implicated in certain diseases, but knockout mice grow and develop normally and are viable [28], [44]. Similarly, *Nrf2* knockout mice are viable and adults exhibit no gross abnormalities, while these animals are hypersensitive to oxidants [45], [46]. Mice lacking *PERK* also develop normally and are viable [47]. All these knockout studies demonstrate that the above genes are largely dispensable for normal growth and development of mice, and that progressive development of certain diseases is only observed later during the life of these mutant animals. There are currently no data regarding the effects of transient inhibition of these processes, with the exception of the non-specific lysosomal degradation inhibitor chloroquine, originally approved for the treatment of malaria, which is already used in the clinic for certain types of cancer [8], [16].

Based on these knockout mouse data, UPR, autophagy and antioxidant responses may be considered as potential non-oncogene addiction pathways: strictly required for Myc-dependent overgrowth (our study) and tumor formation [30], [32], [40], but dispensable for the growth and viability of normal cells, both in Drosophila and mammals. One can speculate that the transient inactivation of these pathways will have even more subtle effects than those observed in knockout mice, but this needs experimental testing. While it is difficult to extrapolate data obtained in Drosophila (or even mouse) studies to human patients, it is tempting to speculate that specific drugs targeting UPR, autophagy and antioxidant responses may prove effective against Myc-dependent human cancers, perhaps without causing adverse side-effects such as current, less specific therapeutic approaches. Notably, widely used anticancer chemotherapy treatments are known to greatly increase the risk that cancer survivors will develop secondary malignancies [48], [49]. Moreover, the autophagy and antioxidant pathways appear to be required in parallel during Myc-induced overgrowth in Drosophila cells. If a similar genetic relationship exists in Myc-dependent human cancer cells, then increased efficacy may be predicted for the combined block of key enzymes acting in these processes.

Elucidation of the genetic alterations behind increased UPR, autophagy and antioxidant responses observed in many established human cancer cells may allow specific targeting of these pathways, and potentially have a tremendous benefit for personalized therapies. In addition to non-specific autophagy inhibitors such as chloroquine, new and more specific inhibitors of selected Atg proteins are being developed [8]. Given the dual roles of autophagy during cancer initiation and progression, a major question is how to identify patients who would likely benefit from taking these drugs. For example, no single test can reliably estimate autophagy levels in clinical samples, as increases in autophagosome generation or decreases in autophagosome maturation and autolysosome breakdown both result in accumulation of autophagic structures [16]. Based on our data and recent mammalian reports, elevated Myc levels may even turn out to be useful as a biomarker before therapeutic application of inhibitors for key autophagy, UPR or antioxidant proteins in cancer patients.

## Materials and Methods

### Drosophila genetics

Flies were maintained on standard cornmeal-yeast-agar medium. We mostly used clonal analysis throughout our work, which represents a very powerful technique, as genetically manipulated cells are surrounded by normal cells in the very same tissue that serve as an internal control, thus eliminating much of the variability that may arise from differences between individual animals. Fat body cell clones were spontaneously generated by Flp recombinase-mediated excision of the FRT cassette from *Actin>y+>Gal4* or *Actin>CD2>Gal4* to activate UAS (upstream activating sequence)-dependent transcription of overexpression, dominant-negative and RNAi transgenes. Myc overexpression was mediated in whole fat bodies by *collagen(cg)-Gal4* for western blots and electron microscopy and by *heat shock(hs)-Gal4* for measuring the time course of autophagy and antioxidant responses, by *patched(ptc)-Gal4* in a stripe of wing imaginal discs that gives rise to the L3–L4 intervein area of adult wings, and by *apterous(ap)-Gal4* in apical epithelial sheets of the developing wings. Fat bodies were dissected from L3 stage larvae 84–90 hours after egg laying, with the exception of Myc mutants and their heterozygous siblings (used as control) when L1 larvae were used as Myc mutants do not develop beyond this stage. Wing discs were dissected from wandering stage L3 larvae (108–120 hours after egg laying), and adult wings were evaluated in 3–7 day old imagoes. The following stocks were used in this study: *Keap1[i1, KK107052], p62[i1, KK108193], cnc[i1, KK108127], FIP200[i, KK104864], Atg18a[i, KK105366], Atg1[i, GD16133], PERK[i, GL00030]* (obtained from the Vienna Drosophila RNAi Center), *p62[i3, HMS00938], p62[i2, HMS00551], cnc[i2, JF02006], cnc[i3, HMS00650], Atg9[i, JF02891], Myc[i, JF01761], Maf[i, JF02008], UAS-Keap1[/2, EY15427], UAS-cnc[EY17502], UAS-Myc* (inserted on chromosome 2), *w[1118]* (control), *ap-Gal4, cg-Gal4,hs-Gal4* (obtained from the Bloomington Drosophila Stock Center), *Myc[4], UAS-Mad* (kindly provided by Peter Gallant) [14], *UAS-Keap1[/1], Keap1[i2, dsRNA], gstD-GFP, gstD-LacZ* (kindly provided by Dirk Bohmann) [50], *UAS-mGadd34, UAS-PERK* (kindly provided by Stefan Marciniak) [35], *ptc-Gal4* (kindly provided by József Mihály), *UAS-Myc* (inserted on chromosome 3, kindly provided by Tom Neufeld), *UAS-mCherry-GFP-Atg8a* [19], *UAS-Xbp1-GFP[HG]* (kindly provided by Hyung Don Ryoo) [36], *UAS-Atg1[DN]* [2], *UAS-Vps34[DN]* [15], *Atg8a[d4]* [17], *FIP200[d130]* (to be described elsewhere). Fat body cell clones expressing *UAS-Myc* were generated by *hs-Flp[22]; UAS-Dcr2; Actin>CD2>Gal4, UAS-GFPnls, r4-mCherry-Atg8a* [17] or by *hs-Flp[22]; UAS-LAMP1-GFP; Actin>CD2>Gal4, UAS-Dcr2* (for Lysotracker and anti-Atg8a stainings) [17]. For epistasis analyses, we used *hsFlp[22]; Actin>y+>Gal4, UAS-GFPnls; UAS-Myc* for all fat body clones with the exception of *FIP200* mutants for which we used *hs-Flp[22], Actin>CD2>Gal4, UAS-mCD8-GFP* (all recombined together on chromosome X), and *ptc-Gal4, UAS-mCherry-Atg8a, UAS-Myc* (all recombined together on chromosome 2) for wing experiments.

### Molecular cloning, cell culture and immunoprecipitation

Coding sequences were PCR amplified for Drosophila p62 (lacking the PB1 domain to prevent self-aggregation), and cloned into pUAST-3×FLAG. Full-length Atg9 and Keap1 were amplified and cloned into pUAST-3×HA. D.Mel-2 cells were maintained in Express Five Serum-Free Medium with penicillin and streptomycin (Invitrogen). Cells were transfected with equal amounts of metallothionein-Gal4, UAS-FLAG-p62 and UAS-HA-Atg9 or UAS-HA-Keap1 plasmids using TransIT-2020 (Mirus). 48 h later, protein expression was induced by 1 mM CuSO_4_ for overnight incubation. Cells were collected by centrifugation, washed twice in PBS and lysed on ice in lysis buffer (0.5% Triton-X100, 150 mM NaCl, 1 mM EDTA, 20 mM TRIS-HCl pH 8) with complete protease and phosphatase inhibitor cocktails (Sigma), spun for 10 min at 10,000 g in an Eppendorf 5430R at 4°C followed by the addition of 30 µl anti-FLAG slurry (Sigma) to the cleared supernatant. After incubation at 4°C for 2 h, beads were collected by centrifugation at 5,000 g for 30 sec at 4°C followed by extensive washes in lysis buffer, and finally boiled in 30 µl Laemmli sample buffer.

### Western blots and immunostainings

50 larval fat bodies per genotype were dissected from well-fed L3 stage larvae, homogenized in Laemmli sample buffer (Sigma), boiled and equal amounts loaded on SDS-PAGE gels, followed by processing as described [4], [17], [19]. Phosphatase inhibitor cocktail PhosSTOP (Roche) was added to samples processed for phospho-eIF2α western blots. Immunostaining of dissected larvae was performed as before [2], [15], [17], [19]. The following primary antibodies were used: rat anti-mCherry (WB 1∶5,000), rat anti-Atg8a (IF 1∶300) [19], rabbit anti-Atg8a (WB 1∶5,000) [19], rabbit anti-p62 (IF 1∶2,000, WB 1∶5,000) [17], mouse anti-Tubulin (WB 1∶1,000, clone AA4.3-s, DSHB), mouse anti-LacZ (IF 1∶100, clone 40-1a-s, DSHB), chicken anti-GFP (IF 1∶1,500, Invitrogen), rabbit anti-phospho-eIF2α (IF 1∶100, WB 1∶1,000, Cell Signaling Technologies), rabbit anti-ubiquitin (1∶500, DAKO). Secondary antibodies were alkaline phosphatase-conjugated goat anti-mouse (WB 1∶ 5,000, Millipore), rabbit anti-rat (WB 1∶ 5,000, Sigma), and Alexa 488 goat anti-chicken, Alexa 546 goat anti-rabbit, Alexa 568 goat anti-rat, Alexa 568 goat anti-mouse (IF 1∶1,500, Invitrogen).

### Histology and microscopy

Approximately 20 larvae were allowed to feed on food supplemented with plenty of dry yeast until reaching the appropriate age. For drug treatment, L1 larvae were transferred to food containing 3 mg/ml chloroquine (Sigma). Starvations were carried out in 20% sucrose solution for 3 hours. Lysotracker and cortical actin Alexa 633-phalloidin (Invitrogen) stainings were carried out as described [2], [15], [17], [19]. All preparations were mounted in 50% glycerol/PBS with 0.2 µM DAPI and viewed on a Zeiss Axioimager M2 equipped with an Apotome2 grid confocal unit, using Plan-NeoFluar 20× 0.5 NA and 40× 0.75 NA objectives, Axiocam Mrm camera, and Axiovision software. DAPI-stained DNA is pseudocoloured red to enhance visibility for actin stainings. Electron microscopy was carried out as before [4], [15], [17]. Adult wings were photographed on a Zeiss Lumar stereomicroscope with Axiocam Icc camera and Axiovision software.

### Image analysis

Original, unmodified images were evaluated in ImageJ (NIH) as before [17], [19]. For fat body measurements, we quantified cell size by manually encircling a GFP-marked cell in ImageJ (NIH) with its nucleus in the focal plane to ensure that the maximal diameter of the given cell is counted, and randomly selected a non-GFP control cell from the very same image (with its nucleus also in the focal plane) to compare it to. The ratio of the GFP-positive cell's size relative to its randomly selected neighbor is calculated as one data point. The only factor we considered was that cells situated at the edge of fat body are usually bigger, so when quantifying a GFP-positive cell at the edge of a fat body lobe we always selected a control cell from the edge as well. Dot number per randomly selected area (400×400 pixel) was measured in the case of Myc null mutant fat bodies and controls, or per whole wing discs, by setting the appropriate threshold in the relevant channel in ImageJ so that as many dots were selected as possible without selecting background fluorescence or merging adjacent separate dots. For clonal analyses, dot number per cell was quantified by first setting the threshold for the relevant channel, and then encircling a GFP-positive cell and recording dot number and size values, followed by evaluating a randomly selected neighboring control cell from the same image, similar to cell size measurements. Dot number per cell data points were corrected according to differences in cell size by normalizing each GFP-positive cell relative to the size of the neighboring control cell, as described [2], [18]. That is, if a Mad overexpressing cell had 2 endogenous Atg8-positive puncta and the size of this cell was 25% of the control cell size, the normalized Atg8 dot number per cell would be 8. Note that inhibition of Myc signaling decreases both cell size and autophagy, meaning that the difference in autophagic dot number between Myc loss of function cells and control cells decreases during normalization (but remains statistically significant). On the other hand, Myc overexpression increases both cell size and autophagy in fat body cells, meaning that the difference in autophagic dot number between Myc gain of function cells and controls again decreases during normalization (but is still statistically very significant). In autophagic flux measurements with and without chloroquine, primary images from mCherry-GFP-Atg8a experiments were analyzed automatically in ImageJ (NIH) with the colocalization plugin using the intensity correlation function [19]. For quantifying *gstD-GFP* expression in fat bodies, the pixel intensity of the green channel was determined in images captured with the same exposure time throughout the experiments.

Western blots were digitized with an Epson Photo 4990 scanner at 600 dpi, by preserving the background. Densitometric evaluation of blots was carried out in ImageJ (NIH). After rectangular selection of individual lanes of interest, these were evaluated using Analyze>Gels>Plot lanes. Then, the peaks of interest were measured in plots by first setting the background with the straight line tool, and then measuring the area under the peak with the wand tool. Values were calculated relative to Tubulin, and fold changes relative to lanes with control lysates are shown in panels.

### Statistics

Sample number (n) in figure legends and Table S1 refers to the number of individual animals analyzed per genotype. For mosaic experiments in fat body cells, 3 GFP versus non-GFP cell pairs were evaluated per animal, with each pair chosen from the same image as described in the image analysis section. For wing disc and adult wing measurements, one disc or wing per animal was quantified. Data were imported into SPSS Statistics version 20 (IBM) and analyzed as indicated in Table S1. For each figure, the table lists genotype for relevant panels, sample number (n), median (or mean), standard deviation, and p value calculated either by two-tailed two-sample unequal variance Student's t test for normal distribution data or by Mann-Whitney u test for non-normal distribution data in pairwise comparisons. We used ANOVA for multiple comparisons of normal distribution data and Kruskal-Wallis test for non-normal distribution data. p<0.05 is considered statistically significant (marked by *) and p<0.01 statistically highly significant (marked by **), and non-significant p values are highlighted by a grey background in the table. Boxplots in figures show median scores, 25% interquartiles, maximum and minimum values, and were also created using SPSS Statistics 20 (IBM). Bar charts for electron microscopy analysis and for time course experiments show average values and standard deviations.

## Supporting Information

Figure S1 Myc overexpression induces autophagy in Drosophila. (A) No autophagy is detected in fat body cells of well-fed control larvae. (B) Myc expression leads to the appearance of double-membrane autophagosomes (ap) containing undigested cytoplasmic material, and dense autolysosomes (al) degrading sequestered cytoplasmic cargo.
(TIF)

Figure S2 Myc-induced overgrowth is suppressed by inhibition of autophagy. (A) Overexpressing two copies of *UAS-Myc* in fat body cell clones, marked by GFP, increases growth compared to neighboring control cells. (B) Chloroquine treatment prevents overgrowth driven by two copies of *UAS-Myc*.(C) Quantification of data in A and B, n = 10–15 per genotype. (D–K) Myc-driven overgrowth of GFP-positive fat cells compared to surrounding control cells is inhibited by *Atg1* RNAi (D), expression of dominant-negative Atg1 (E) or Vps34 (F), silencing of *Atg18a* (G), *Atg9* (H), *FIP200* (I), *Myc* (J), or overexpression of Mad (K). (L) In adult wings, the *patched* domain corresponds to the region between longitudinal veins L3 and L4 (marked by mCherry-Atg8a expression in lower panel). Wing vein distance was quantitated based on the ratio of L3–L4 distance (measured halfway between PCV and wing margin) to L4–L5 vein distance at the posterior crossvein (PCV) for each individual wing, as indicated in the upper panel. (M–O) Myc-induced punctate mCherry-Atg8a labeling and expansion of the patched expression domain is inhibited by depletion of *FIP200* (M) or *Atg18a* (N), or by expression of dominant-negative Vps34 (O). (P) Silencing of *FIP200, Atg1, Atg9, Atg18a* or expression of dominant-negative Vps34 has no effect on the growth of fat body cells. Expression of dominant-negative Atg1 reduces cell size, likely due to competition of the overexpressed catalitically inactive protein with other substrates of TOR kinase, such as the critical growth regulator S6K. Depletion of *Myc* or Mad overexpression also reduces cell size. n = 7 for all genotypes. (Q) L3–L4 wing vein distance is not reduced compared to control wings upon inhibition of *Atg1*, *FIP200*, *Vps34* or *Atg18a*, n = 13–21 per genotype. Scalebars in A, B, D–K, M–O equal 20 µm. Statistically significant differences are indicated, ** p<0.01.
(TIF)

Figure S3 Myc-induced overgrowth of the apical epithelial sheet in the wing is blocked by inhibition of autophagy or *p62*. (A) The wings of control flies are straight. (B) Myc overexpression in the *apterous* expression domain results in overgrowth of the apical epithelial layer, producing flies with downward curving wings. (C–F) The downward-curving phenotype of Myc overexpressing wings is suppressed by null mutation of *Atg8a* (C), expression of dominant-negative Vps34 (D), depletion of *Atg18a* (E) or *p62* (F).
(TIF)

Figure S4 Myc-induced overgrowth requires antioxidant responses. (A) No expression of the Nrf2/cnc-dependent transcriptional reporter gstD-GFP is seen in the *patched* expressing domain (marked by mCherry-Atg8a) in control wing imaginal discs. (B) Myc overexpression induces gstD-GFP expression in the mCherry-Atg8a expressing domain. (C, D) Knockdown of *p62* or *cnc* prevents activation of GstD-GFP by Myc. (E–K) Myc-induced overgrowth of GFP-positive cells compared to neighboring control cells is inhibited by depletion of *p62* (E, F), *cnc* (G–I), *Maf* (J), or overexpression of Keap1 (K). (L, M) GFP-positive cells overexpressing Myc remain much bigger than control cells upon *Keap1* depletion (L) or overexpression of cnc (M). (N) *Keap1* depletion restores Myc-induced overgrowth in *p62* RNAi cells. (O, P) Overexpression of cnc fails to restore Myc-induced overgrowth in *FIP200* RNAi (O) or dominant-negative Vps34 expressing cells (P). (Q) Modulation of p62/cnc signaling in GFP-positive cell clones has no effect on the size of these fat body cells relative to neighboring control cells. n = 7 for all genotypes. (R) Modulation of p62/cnc signaling has no or minor effects on L3–L4 wing vein distance in adult wings, n = 12–19 per genotype. (S, T) Depletion of *p62* (S) or overexpression of Keap1 (T) reduces the area of the Myc-expressing *patched* domain but does not block punctate mCherry-Atg8a labeling. Scalebars in A–P and S, T equal 20 µm. Statistically significant differences are indicated, * p<0.05 and ** p<0.01.
(TIF)

Figure S5 Temporal regulation of autophagy and Nrf2 activity by Myc. (A) Induction of Myc expression by a 2-hour heat shock results in the formation of numerous mCherry-GFP-Atg8a punctae by 12 and 18 hours after induction. Practically no dots are seen in fat body cells of control larvae upon heat shock-mediated expression of mCherry-GFP-Atg8a. n = 12 for each genotype/time point. (B) Expression of the Nrf2-dependent transcriptional reporter *gstD-GFP* is similar to basal fat body expression levels (not shown) at 4 hours after heat shock-mediated induction of Myc and in control larvae expressing only *hs-Gal4*. Upregulation of this reporter becomes obvious by 12 and 18 hours after induction of Myc. n = 10 for each genotype/time point. Scalebars equal 20 µm. Statistically significant differences are indicated, ** p<0.01.
(TIF)

Figure S6 PERK signaling is largely dispensable for cell growth. (A) The unfolded protein response (UPR) reporter XBP1-GFP shows only diffuse fluorescnce in control wing discs that express mCherry-Atg8a in the *patched* domain. (B) Xbp1-GFP expression is enhanced by overexpression of Myc in the mCherry-Atg8a expression area. (C) Depletion of *PERK* or overexpression of its antagonist Gadd34 in GFP-positive cell clones has no effect on the size of these fat body cells relative to neighboring control cells. n = 7 for both genotypes. (D) *PERK* RNAi or Gadd34 overexpression only slightly reduces L3–L4 vein distance compared to L4–L5 vein distance in adult wings, n = 12–19 per genotype. Scalebars in A, B equal 20 µm. Statistically significant differences are indicated, ** p<0.01.
(TIF)

Table S1 Quantification of experimental data. The number of individual animals quantified (n) is indicated for all genotypes. Note that in mosaic analyses, clone and control cell pairs were always evaluated from the same image, same tissue, same animal. P values≥0.05 (considered not statistically significant) are highlighted by a grey background. Please see methods for further details.
(XLSX)
