# Supplementary figures and images for: Myc-Driven Overgrowth Requires Unfolded Protein Response-Mediated Induction of Autophagy and Antioxidant Responses in Drosophila melanogaster
Source: PLoS Genet. 2013 Aug 8;9(8):e1003664. doi: 10.1371/journal.pgen.1003664 (PMC3738540; doi:10.1371/journal.pgen.1003664)

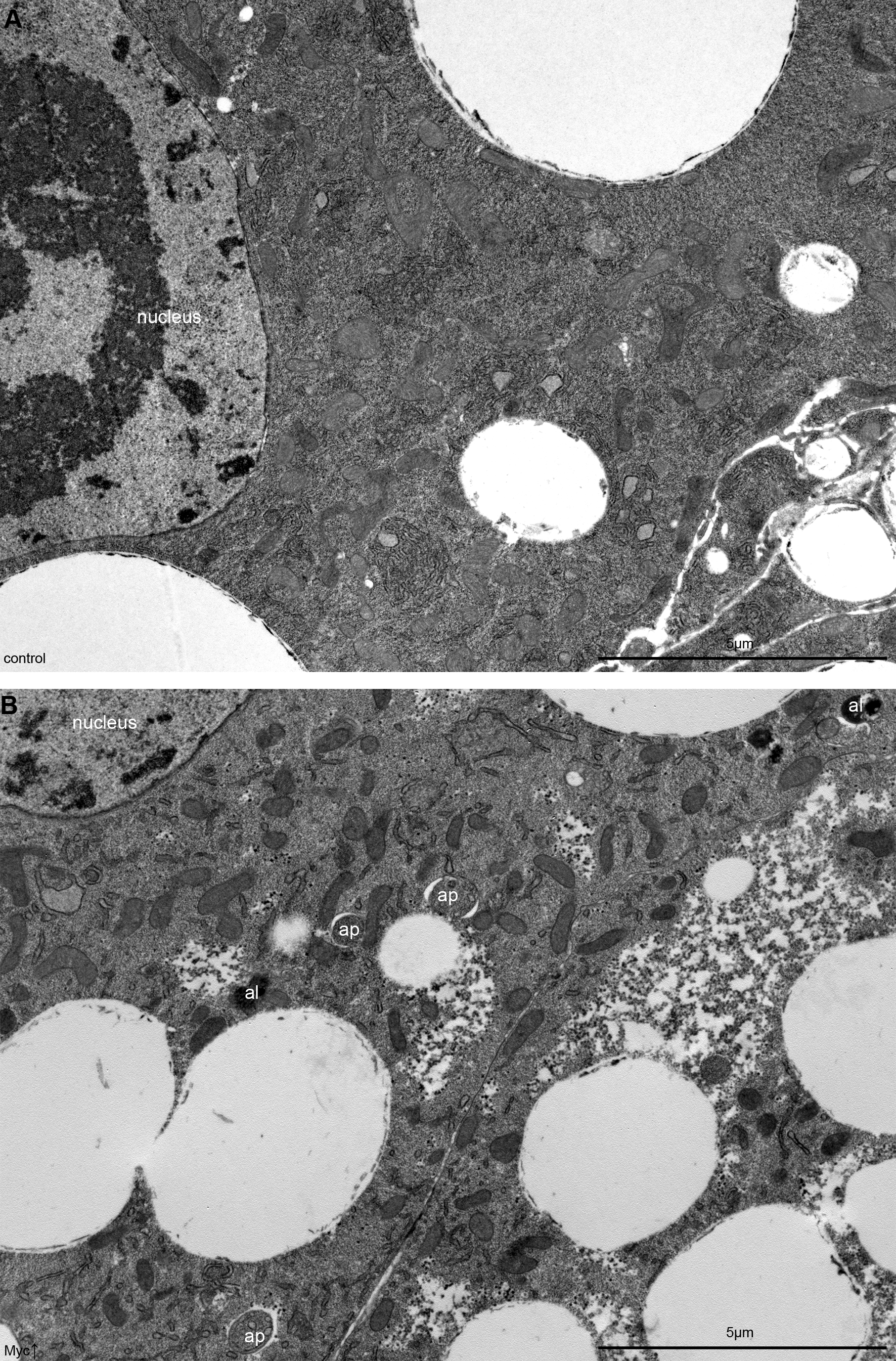

Supplement: Figure S1 — Myc overexpression induces autophagy in Drosophila. (A) No autophagy is detected in fat body cells of well-fed control larvae. (B) Myc expression leads to the appearance of double-membrane autophagosomes (ap) containing undigested cytoplasmic material, and dense autolysosomes (al) degrading sequestered cytoplasmic cargo. (TIF) [file pgen.1003664.s001.tif]

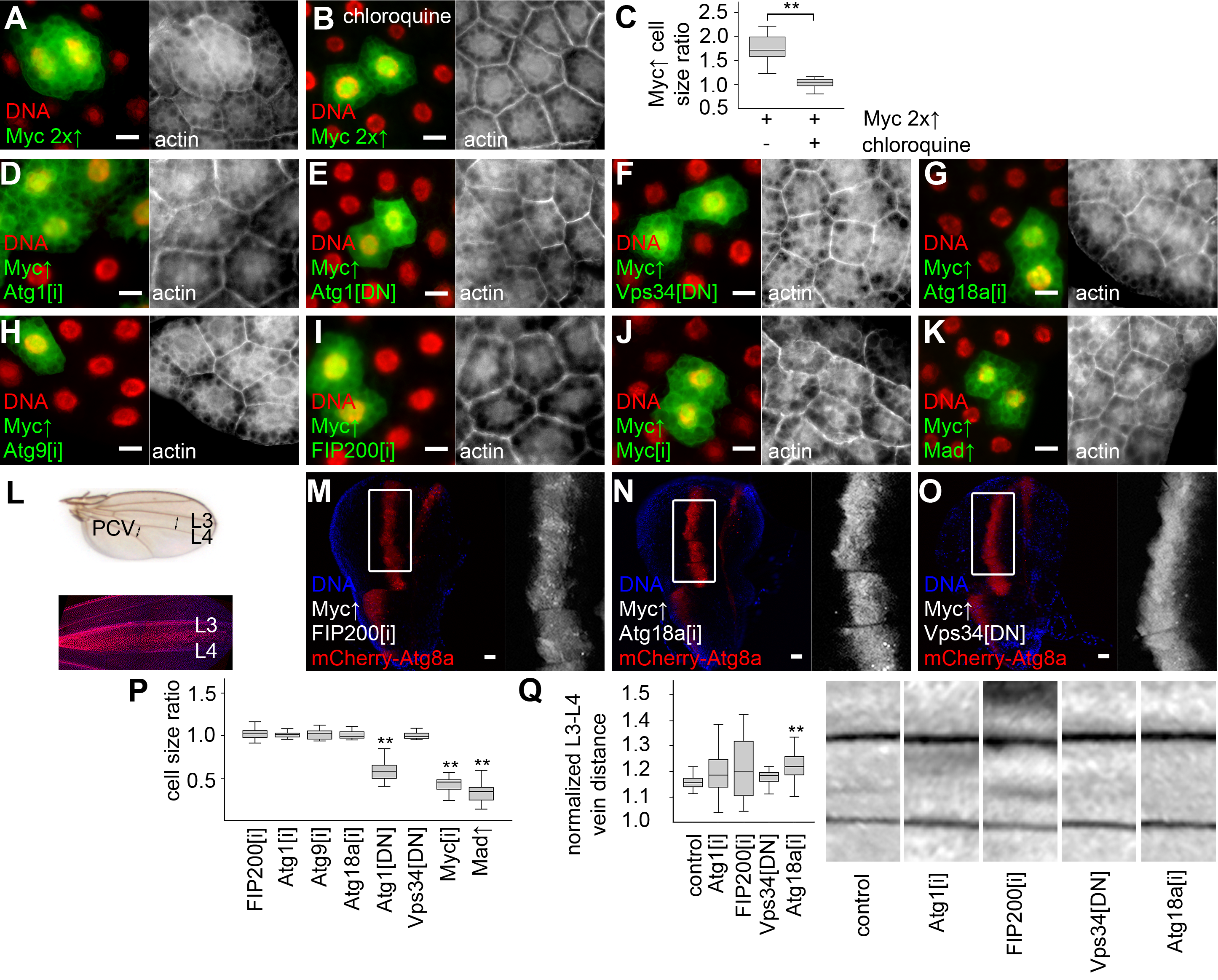

Supplement: Figure S2 — Myc-induced overgrowth is suppressed by inhibition of autophagy. (A) Overexpressing two copies of UAS-Myc in fat body cell clones, marked by GFP, increases growth compared to neighboring control cells. (B) Chloroquine treatment prevents overgrowth driven by two copies of UAS-Myc.(C) Quantification of data in A and B, n = 10–15 per genotype. (D–K) Myc-driven overgrowth of GFP-positive fat cells compared to surrounding control cells is inhibited by Atg1 RNAi (D), expression of dominant-negative Atg1 (E) or Vps34 (F), silencing of Atg18a (G), Atg9 (H), FIP200 (I), Myc (J), or overexpression of Mad (K). (L) In adult wings, the patched domain corresponds to the region between longitudinal veins L3 and L4 (marked by mCherry-Atg8a expression in lower panel). Wing vein distance was quantitated based on the ratio of L3–L4 distance (measured halfway between PCV and wing margin) to L4–L5 vein distance at the posterior crossvein (PCV) for each individual wing, as indicated in the upper panel. (M–O) Myc-induced punctate mCherry-Atg8a labeling and expansion of the patched expression domain is inhibited by depletion of FIP200 (M) or Atg18a (N), or by expression of dominant-negative Vps34 (O). (P) Silencing of FIP200, Atg1, Atg9, Atg18a or expression of dominant-negative Vps34 has no effect on the growth of fat body cells. Expression of dominant-negative Atg1 reduces cell size, likely due to competition of the overexpressed catalitically inactive protein with other substrates of TOR kinase, such as the critical growth regulator S6K. Depletion of Myc or Mad overexpression also reduces cell size. n = 7 for all genotypes. (Q) L3–L4 wing vein distance is not reduced compared to control wings upon inhibition of Atg1, FIP200, Vps34 or Atg18a, n = 13–21 per genotype. Scalebars in A, B, D–K, M–O equal 20 µm. Statistically significant differences are indicated, ** p<0.01. (TIF) [file pgen.1003664.s002.tif]

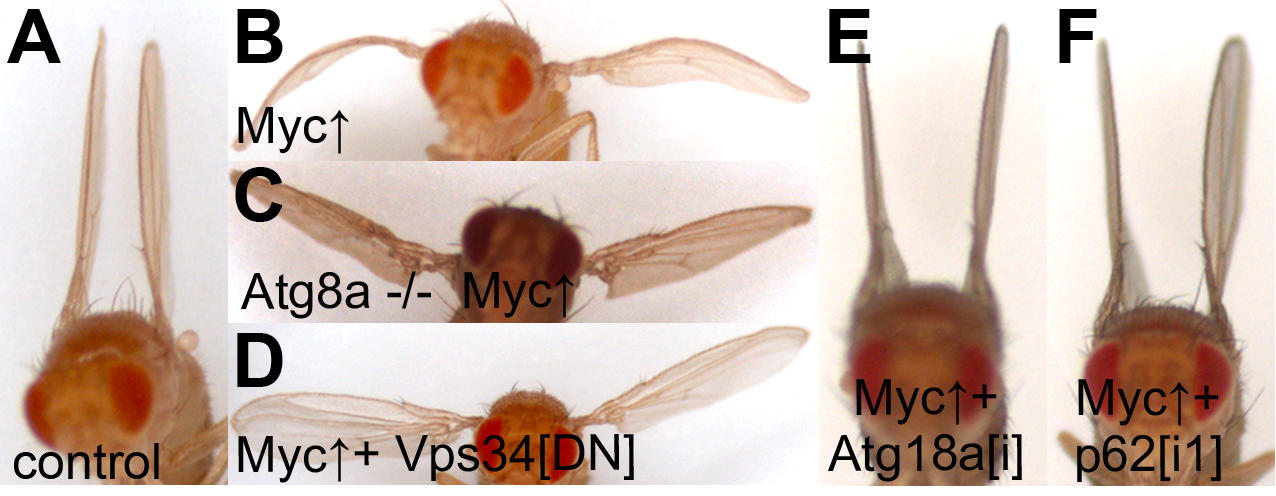

Supplement: Figure S3 — Myc-induced overgrowth of the apical epithelial sheet in the wing is blocked by inhibition of autophagy or p62. (A) The wings of control flies are straight. (B) Myc overexpression in the apterous expression domain results in overgrowth of the apical epithelial layer, producing flies with downward curving wings. (C–F) The downward-curving phenotype of Myc overexpressing wings is suppressed by null mutation of Atg8a (C), expression of dominant-negative Vps34 (D), depletion of Atg18a (E) or p62 (F). (TIF) [file pgen.1003664.s003.tif]

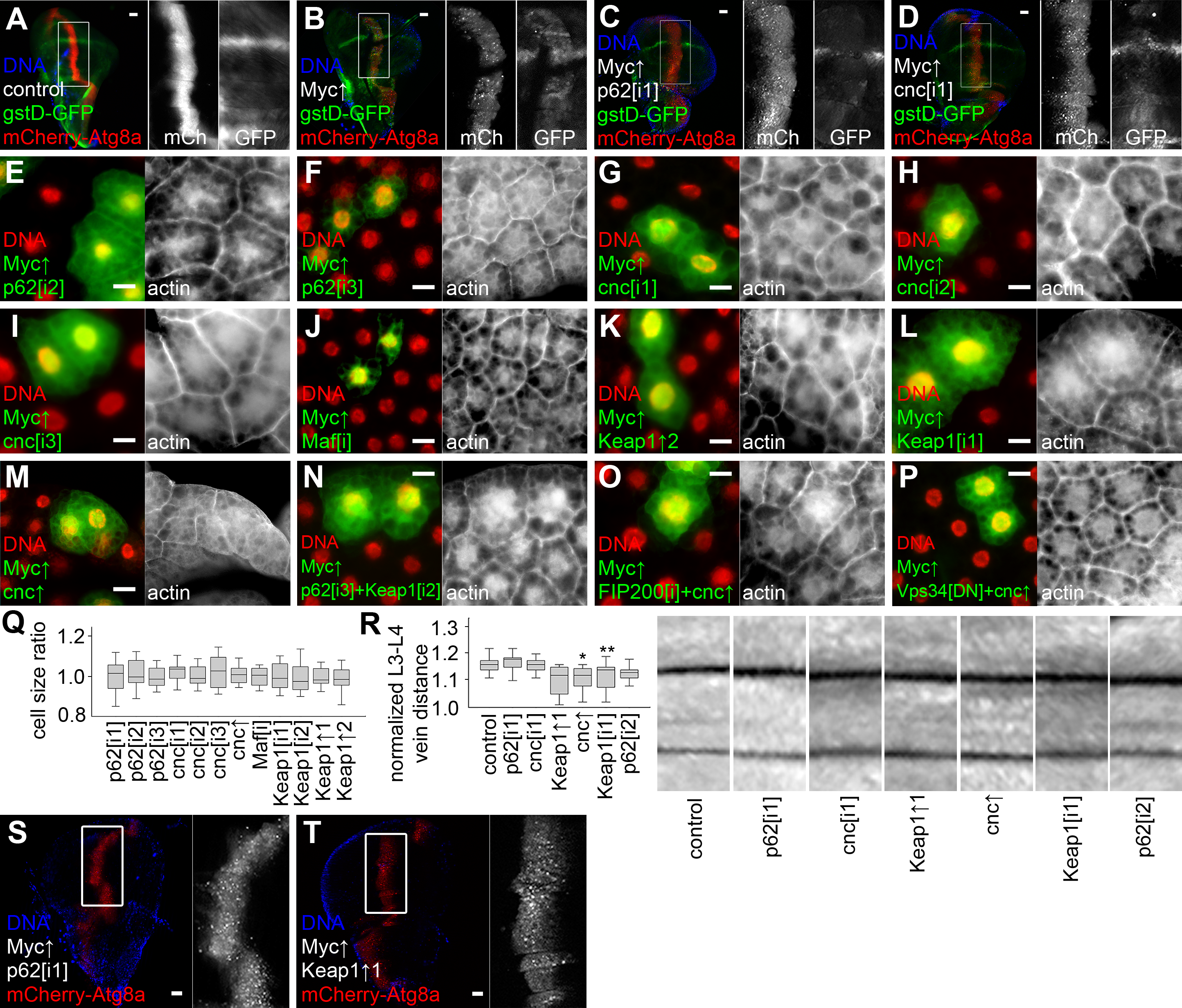

Supplement: Figure S4 — Myc-induced overgrowth requires antioxidant responses. (A) No expression of the Nrf2/cnc-dependent transcriptional reporter gstD-GFP is seen in the patched expressing domain (marked by mCherry-Atg8a) in control wing imaginal discs. (B) Myc overexpression induces gstD-GFP expression in the mCherry-Atg8a expressing domain. (C, D) Knockdown of p62 or cnc prevents activation of GstD-GFP by Myc. (E–K) Myc-induced overgrowth of GFP-positive cells compared to neighboring control cells is inhibited by depletion of p62 (E, F), cnc (G–I), Maf (J), or overexpression of Keap1 (K). (L, M) GFP-positive cells overexpressing Myc remain much bigger than control cells upon Keap1 depletion (L) or overexpression of cnc (M). (N) Keap1 depletion restores Myc-induced overgrowth in p62 RNAi cells. (O, P) Overexpression of cnc fails to restore Myc-induced overgrowth in FIP200 RNAi (O) or dominant-negative Vps34 expressing cells (P). (Q) Modulation of p62/cnc signaling in GFP-positive cell clones has no effect on the size of these fat body cells relative to neighboring control cells. n = 7 for all genotypes. (R) Modulation of p62/cnc signaling has no or minor effects on L3–L4 wing vein distance in adult wings, n = 12–19 per genotype. (S, T) Depletion of p62 (S) or overexpression of Keap1 (T) reduces the area of the Myc-expressing patched domain but does not block punctate mCherry-Atg8a labeling. Scalebars in A–P and S, T equal 20 µm. Statistically significant differences are indicated, * p<0.05 and ** p<0.01. (TIF) [file pgen.1003664.s004.tif]

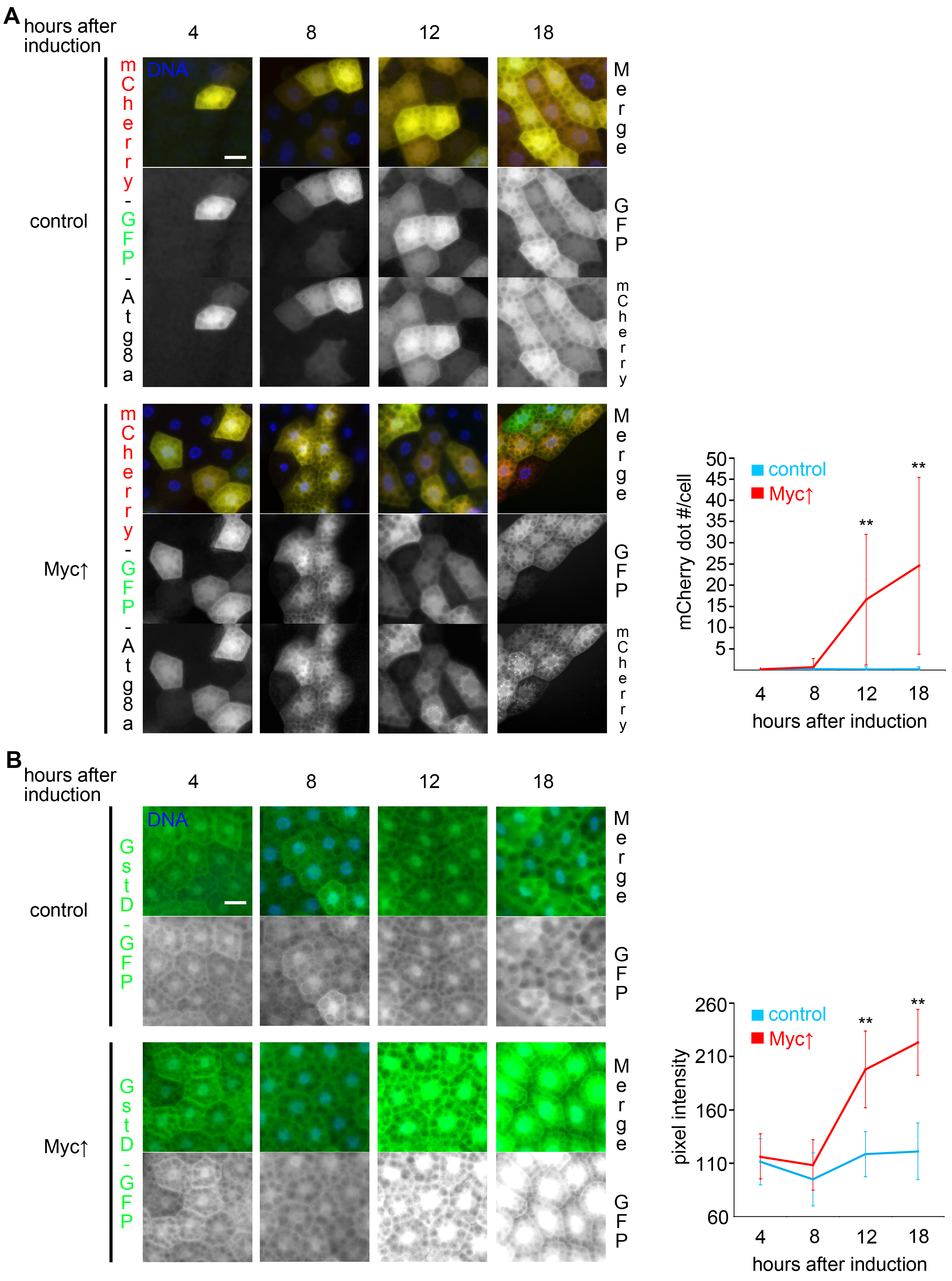

Supplement: Figure S5 — Temporal regulation of autophagy and Nrf2 activity by Myc. (A) Induction of Myc expression by a 2-hour heat shock results in the formation of numerous mCherry-GFP-Atg8a punctae by 12 and 18 hours after induction. Practically no dots are seen in fat body cells of control larvae upon heat shock-mediated expression of mCherry-GFP-Atg8a. n = 12 for each genotype/time point. (B) Expression of the Nrf2-dependent transcriptional reporter gstD-GFP is similar to basal fat body expression levels (not shown) at 4 hours after heat shock-mediated induction of Myc and in control larvae expressing only hs-Gal4. Upregulation of this reporter becomes obvious by 12 and 18 hours after induction of Myc. n = 10 for each genotype/time point. Scalebars equal 20 µm. Statistically significant differences are indicated, ** p<0.01. (TIF) [file pgen.1003664.s005.tif]

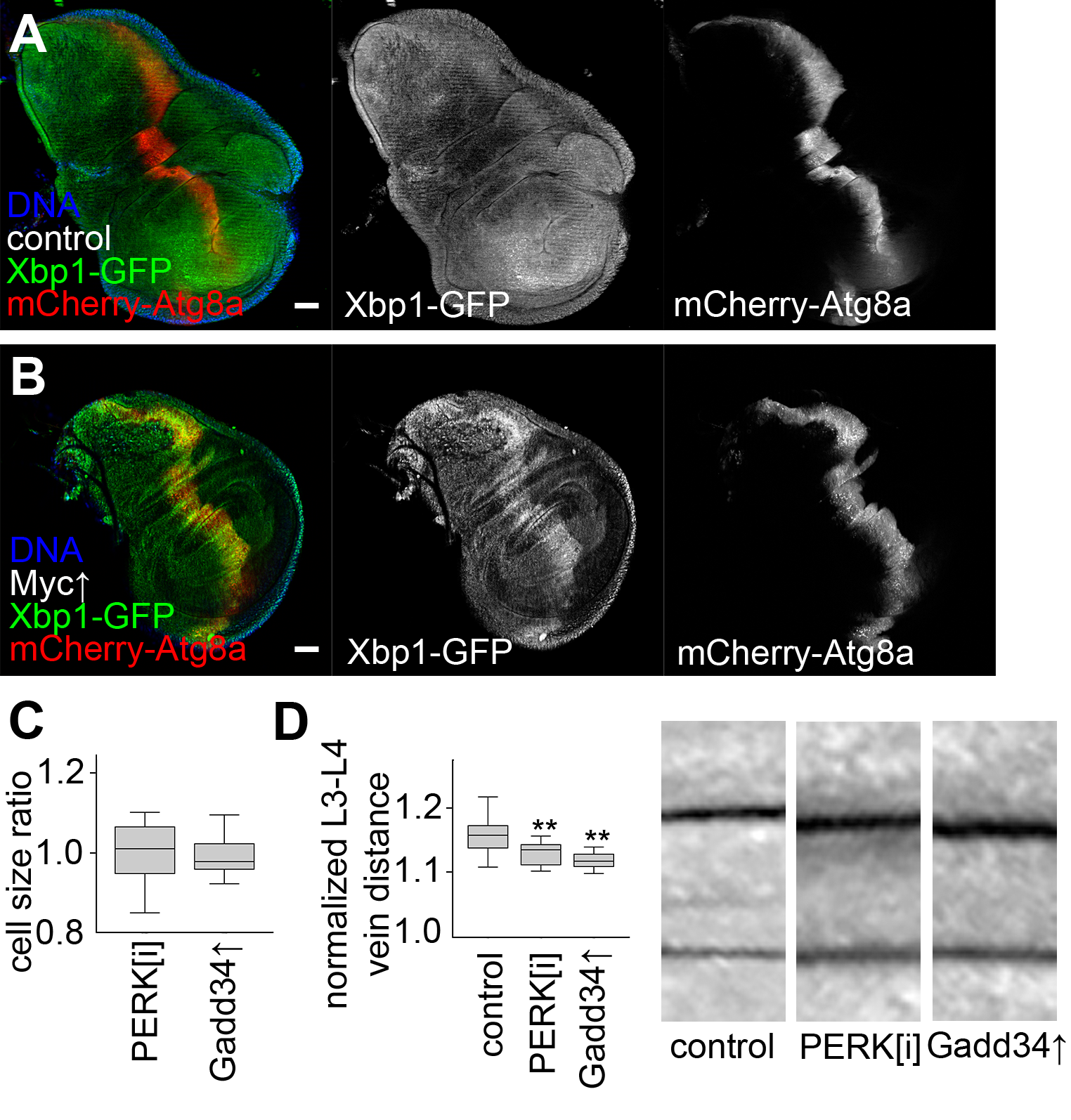

Supplement: Figure S6 — PERK signaling is largely dispensable for cell growth. (A) The unfolded protein response (UPR) reporter XBP1-GFP shows only diffuse fluorescnce in control wing discs that express mCherry-Atg8a in the patched domain. (B) Xbp1-GFP expression is enhanced by overexpression of Myc in the mCherry-Atg8a expression area. (C) Depletion of PERK or overexpression of its antagonist Gadd34 in GFP-positive cell clones has no effect on the size of these fat body cells relative to neighboring control cells. n = 7 for both genotypes. (D) PERK RNAi or Gadd34 overexpression only slightly reduces L3–L4 vein distance compared to L4–L5 vein distance in adult wings, n = 12–19 per genotype. Scalebars in A, B equal 20 µm. Statistically significant differences are indicated, ** p<0.01. (TIF) [file pgen.1003664.s006.tif]
